# A new micropolydesmoid millipede of the genus *Eutrichodesmus* Silvestri, 1910 from Cambodia, with a key to species in mainland Southeast Asia (Diplopoda, Polydesmida, Haplodesmidae)

**DOI:** 10.3897/zookeys.996.57411

**Published:** 2020-11-24

**Authors:** Ruttapon Srisonchai, Natdanai Likhitrakarn, Chirasak Sutcharit, Ekgachai Jeratthitikul, Warut Siriwut, Phanara Thrach, Samol Chhuoy, Peng Bun Ngor, Somsak Panha

**Affiliations:** 1 Animal Systematics Research Unit, Department of Biology, Faculty of Science, Chulalongkorn University, Phayathai Road, Patumwan, Bangkok 10330, Thailand Chulalongkorn University Bangkok Thailand; 2 Division of Plant Protection, Faculty of Agricultural Production, Maejo University, San Sai, Chiang Mai 50290, Thailand Maejo University Chiang Mai Thailand; 3 Animal Systematics and Molecular Ecology Laboratory, Department of Biology, Faculty of Science, Mahidol University, Bangkok 10400, Thailand Mahidol University Bangkok Thailand; 4 Inland Fisheries Research and Development Institute (IFReDI), Fisheries Administration, No. 86, Norodom Blvd., PO Box 582, Phnom Penh, Cambodia Inland Fisheries Research and Development Institute Phnom Penh Cambodia; 5 Wonders of the Mekong Project, c/o IFReDI, No. 86, Norodom Blvd., PO Box 582, Phnom Penh, Cambodia Wonders of the Mekong Project Phnom Penh Cambodia; 6 Academy of Science, The Royal Society of Thailand, Bangkok 10300, Thailand Academy of Science, The Royal Society of Thailand Bangkok Thailand

**Keywords:** Karst, new species, Southeast Asia, taxonomy

## Abstract

The micropolydesmoid millipede family Haplodesmidae is here recorded from Cambodia for the first time through the discovery of the first, new species of the genus *Eutrichodesmus* Silvestri, 1910: *E. cambodiensis***sp. nov.** This new species is described from two limestone habitats in Kampot Province, based on abundant material. It is easily distinguished from all related congeners by the following combination of characters: body greyish-brown; limbus roundly lobulate; solenomere partially divided from acropodite by a digitiform lobe, but without hairpad. Brief remarks on the previously-proposed “*pecularis*-group” are provided and a second group, the “*demangei*-group”, is established and discussed on the basis of morphological evidence, updating the number of recognised species groups of *Eutrichodesmus* to two. Detailed morphological illustrations, photographs and a distribution map, as well as remarks on its habitat and mating behaviour of the new species are presented. Furthermore, the current distributions of all 55 presently-known species of *Eutrichodesmus* are provided and a key to all 23 species that occur in mainland Southeast Asia is given.

## Introduction

Previous and recent studies on millipedes in the Kingdom of Cambodia have revealed at least 23 species from 17 genera, 12 families and eight orders (Likhitrakarn et al. 2015, 2020; Golovatch 2018). Although the Polydesmida Pocock, 1887 is the most diverse order of Diplopoda worldwide, only two polydesmidan families have hitherto been reported from Cambodia: Cryptodesmidae and Paradoxosomatidae. The Cryptodesmidae is typically considered as “micropolydesmoid” due to small body sizes of its species. The group is represented in Cambodia by only two species: *Trichopeltis kometis* (Attems, 1938) and *Circulocryptus kompantsevi* Golovatch, 2018. The former species was originally described from Kratié Province (Attems 1938), but it has since been recorded from Vietnam and Laos as well (Golovatch and Akkari 2016). *Circulocryptus kompantsevi* has recently been described from a rain–and-cloud forest at about 1,000 m above sea-level (AMSL) in the Phnom Bokor National Park, Kampot Province (Golovatch 2018). Surprisingly, no other micropolydesmoid families (e.g. Haplodesmidae or Pyrgodesmidae), both quite diverse and common in Indochina, have been recorded from Cambodia yet. Not only the micropolydesmoids, but also the genera *Desmoxytes* Chamberlin, 1923, *Antheromorpha* Jeekel, 1968 and *Tylopus* Jeekel, 1968, all in the family Paradoxosomatidae and all quite diverse and common in the neighbouring countries, also appear to be poorly represented in Cambodia. This strongly contrasts with the adjacent parts of Indochina where more than 20 micropolydesmoid species have been discovered in Laos, Thailand and Vietnam over the last few years (Golovatch et al. 2015; Liu et al. 2017; Golovatch 2018; Likhitrakarn et al. 2019). Amongst these micropolydesmoids, many are quite rare and most are known only from their original descriptions.

The micropolydesmoid genus *Eutrichodesmus* Silvestri, 1910 is amongst the most speciose not only in Haplodesmidae, but also in the entire order Polydesmida. Its distribution ranges from southern Japan in the north, through Taiwan, continental China and mainland Southeast Asia, to Indonesia and Vanuatu in the south (Liu and Wynne 2019; Golovatch and Liu 2020). *Eutrichodesmus* currently comprises 54 recognised species (Sierwald and Spelda 2019), of which over half are known from continental China alone, whereas 22 species are restricted to mainland Southeast Asia. Golovatch et al. (2009a, 2009b) provided the most thorough and basic treatment of the group. However, since then, the number of known species has increased almost three-fold (Golovatch et al. 2009b, 2010, 2015, 2016; Makhan 2010; Liu et al. 2013, 2017; Liu and Wynne 2019). This invites an update and a modern key.

We have recently conducted surveys in southern Cambodia with emphasis on the biodiversity of limestone karsts. A considerable amount of material has been collected and become available for study. As a result, several new species from different millipede groups have been revealed and mostly already described: *Plusioglyphiulus* Silvestri, 1923 and *Trachyjulus* Peters, 1864 (Cambalopsidae, Spirostreptida), as well as *Tylopus* and *Orthomorpha* Bollman, 1893 (Paradoxosomatidae, Polydesmida). The present paper is devoted to the description of a new *Eutrichodesmus*, the first Haplodesmidae to be recorded from Cambodia. We also provide an identification key to and update the distributions of all known species of *Eutrichodesmus*.

## Material and methods

The material for this contribution was collected during surveys on freshwater and terrestrial invertebrates in Cambodia, conducted jointly by researchers from the Inland Fisheries Research & Development Institute of Cambodia (IFReDI) and several Thai specialists. Since the expeditions started (from 2018 until now), large collections of millipedes have become available, also representing the first reference collections in Cambodia.

### Specimen collecting and preservation

All specimens were hand-collected from limestone habitats in Cambodia. Live animals were photographed using a Nikon D700, equipped with an AF-S VR Micro-Nikkor 105 mm lens in the field. Some mating pairs were observed at the type locality and some were brought back to the laboratory for further behavioural observations. Specimens were euthanised, based on AVMA guidelines for the euthanasia of animals (American Veterinary Medical Association 2020) and then mostly stored in 70% (v/v) ethanol for morphological study and, partly, in 95% (v/v) ethanol for molecular analysis. Latitude, longitude and elevation were obtained from a Garmin GPSMAP 60 CSx and all coordinates and elevations were double-checked with Google Earth to confirm the precise location.

### Morphological descriptions

All specimens of the new species were carefully examined for non-gonopodal and gonopodal characteristics using stereo and compound light microscopes. For some male specimens, the gonopods were carefully dissected and then mounted on a slide with DPX/ balsam. The morphological terminology used in this study follows that of previous publications (Golovatch et al. 2009a, 2009b, 2010, 2015, 2016; Hoffman 1977a, 1977b; Liu and Tian 2013; Liu et al. 2017). Details of gonopodal terminology are shown in the section “Abbreviations used in descriptions” below.

The holotype and some paratypes are deposited in the Chulalongkorn University Museum of Zoology (CUMZ–hpd0001 and CUMZ–hpd0002). Some paratypes are housed in the collections of the Inland Fisheries Research and Development Institute (CIFI), Cambodia and the Zoological Reference Collection (ZRC) of the Lee Kong Chian Natural History Museum, Singapore.

All available literature sources, especially the original descriptions, were critically accessed in order to compare morphological characters to all known species. Positional and directional terms for gonopod descriptions follow Srisonchai et al. (2018).

### Illustrations

Drawings were sketched under a stereomicroscope and a light microscope. All plates of figures were generated and edited using Adobe Photoshop CS6 to adjust the colour and brightness. The distribution map was modified from Willett et al. (2015).

### Abbreviations used in descriptions

As the previous studies of gonopods from different authors are quite variable, ranging from a brief description to several deeply detailed ones, we chose to follow the comprehensive gonopod terminology from Golovatch et al. (2009b, 2015), Hoffman (1977a, 1977b) and Liu et al. (2017).

Abbreviations: cn = cannula, cx = coxa, dp = distofemoral process, sg = seminal groove; acropodite = the apical part of gonopod that starts from a prominent cingulum (the end of the femorite); solenomere = an independent part of the gonopod acropodite that carries the seminal groove, with or without hairpad, completely or partly fused to the acropodite; telopodite = the main part of the gonopod pivoting on the coxa, including the prefemur, femorite and acropodite.

#### Other abbreviations used

**AMSL** above mean sea level

**ca.** about, around, circa;

**CIFI** the collection of the Inland Fisheries Research and Development Institute, Cambodia;

**CUMZ**Chulalongkorn University Museum of Zoology, Bangkok, Thailand;

**IFReD** Inland Fisheries Research and Development Institute, Cambodia;

**SEM** Scanning electron microscopy;

**ZRC**Zoological Reference Collection of the Lee Kong Chian Natural History Museum, National University of Singapore, Singapore.

## Results

### Taxonomy

**Order Polydesmida Pocock, 1887**

**Suborder Polydesmidea Pocock, 1887**

#### Family Haplodesmidae Cook, 1895

##### Genus Eutrichodesmus

Taxon classificationAnimaliaPolydesmideaHaplodesmidae

Silvestri, 1910

F63E2E4C-6014-5C68-81DB-F6D8742FF80B

###### Type species.

*Eutrichodesmus demangei* Silvestri, 1910

###### All species included.

The genus *Eutrichodesmus* currently contains 55 species, including the new one described herein, see Table 1.

###### Recorded distributions of all known species.

Based on all the recent literature and excluding the newly-described species, the genus *Eutrichodesmus* is widely distributed in southern Japan, Taiwan, southern China, mainland Southeast Asia (Malaysia, Laos, Thailand and Vietnam), Indonesia (Sulawesi) and Melanesia (Vanuatu) (Golovatch et al. 2009a, 2009b, 2015, 2016; Liu et al. 2017; Golovatch and Liu 2020; see Table 1). No *Eutrichodesmus* species have hitherto been reported from Cambodia.

**Table 1.** Known distribution of all *Eutrichodesmus* species. Note that the species are listed, based on the appearance of mid-dorsal projections on metaterga due to the simpliest way for their examination.

No.	Species	Type locality	Distribution	References
**Species with conspicuous mid-dorsal projections on metaterga**				
1	*E. anisodentus* (Zhang, 1995)	China, Fujian Province, Mt Wuyi	South-eastern China	Zhang 1995b; Golovatch et al. 2010, 2015
2	*E. aster* Golovatch et al., 2009	Vietnam, Yen Bai Province, Nghia Lo: Xa Som a, Tham Han Cave	North-western Vietnam	Golovatch et al. 2009b
3	*E. asteroides* Golovatch et al., 2009	Vietnam, Quang Binh Province, Cha Noi: Hang Cha Noi Cave	Central Vietnam	Golovatch et al. 2009b
4	*E. astriproximus* Golovatch et al., 2016	Vietnam, Quang Binh Province, Thuong Hoa, Cave Hang Mo O	Central Vietnam	Golovatch et al. 2016
5	*E. astrisimilis* Golovatch et al., 2016	Vietnam, Quang Binh Province, Hoan Son, Cave Hang Cha Ra	Central Vietnam	Golovatch et al. 2016
6	*E. cavernicola* (Sinclair, 1901)	Thailand, Yala Province, Mueang Yala District, Wat Khuhapimuk (Gua Glaf = Gua Galp = Dark Cave) (exact location based on Huber et al. (2015))	Southern Thailand	Sinclair 1901; Hoffman 1977b; Golovatch et al. 2009a, 2009b
7	*E. deporatus* Liu & Wesener, 2017	Laos, Luang Prabang Province, Northeast of Luang Prabang, Nam Ou, Nong Khiao, Cave Tham Pathok	Northern Laos	Liu et al. 2017
8	*E. dorsiangulatus* (Zhang *in* Zhang & Wang, 1993)	China, Yunnan Province, Mengla County, Baoniujiao Cave	South-western China	Zhang and Wang 1993; Golovatch et al. 2009a, 2009b, 2015
9	*E. lipsae* Golovatch et al., 2015	China, Guangxi Province, Guilin County, Grotte des Squelettes	Southern China	Golovatch et al. 2015
10	*E. macclurei* (Hoffman, 1977)	Malaysia, Selangor State, near Kuala Lumpur, Batu Caves	Peninlular Malaysia	Hoffman 1977a; Golovatch et al. 2009a, 2009b
11	*E. nodulosus* (Verhoeff, 1939)	Japan, Ryukyu Island of “Fukafuguza”, a cave (Fukafuguza not known to exist in the Ryukyu Island)	Southern Japan	Verhoeff 1939; Omine 1982; Omine and Ito 1998; Golovatch et al. 2010
12	*E. paraster* Liu & Wesener, 2017	Laos, Huaphan Province, Xop, Cave Tham Long Puang	Eastern Laos	Liu et al. 2017
13	*E. pectinatidentis* (Zhang, 1995)	China, Zhejiang Province, Lin’an County, Mt. Tianmu	East-central China	Zhang 1995a; Golovatch et al. 2010, 2015
14	*E. reclinatus* (Hoffman, 1977)	Malaysia, Selangor State, near Kuala Lumpur, Gua Anak Takun at Templer Park	Peninsular Malaysia	Hoffman 1977b; Golovatch et al. 2009a, 2009b
15	*E. soesilae* Makhan, 2010	China, Chongqing Municipality, Beibei District, Mt. Jinyun	South-western China	Makhan 2010; Golovatch et al. 2010, 2015
16	*E. steineri* Liu & Wesener, 2017	Laos, Luang Prabang Province, Phou Khoun District, Cave Tham Deu	Northern Laos	Liu et al. 2017
17	*E. subasteroides* Golovatch et al., 2016	Vietnam, Quang Binh Province, Hoan Son, Cave Hang Da Voi	Central Vietnam	Golovatch et al. 2016
**Species without mid-dorsal projections on metaterga**				
18	*E. apicalis* Golovatch et al., 2015	China, Hubei Province, Yishang Yichang County, Grotte des Araignées	Central China	Golovatch et al. 2015
19	*E. arcicollaris* Zhang *in* Zhang & Wang, 1993	China, Yunnan Province, Hekou County, near Laofanzhai Village, Huayu Cave	South-western China	Zhang and Wang 1993; Golovatch et al. 2009a, 2009b, 2015
20	*E. armatocaudatus* Golovatch et al., 2009	Vietnam, Thanh Hoa Province, Pu Luong, Lung Cao, Hang Lang Lua Cave	Northern Vietnam	Golovatch et al. 2009a
21	*E. armatus* (Miyosi, 1951)	Japan, Ehime Prefecture, Kaminada-Mati, Yosihuzi-Mura (Shikoku Island)	South-western & Southern Japan; Taiwan	Miyosi 1951; Golovatch et al. 2010; Wang 1958; Karasawa et al. 2008
22	*E. basalis* Golovatch et al., 2009	Vietnam, Vinh Ha Long Province (Southwest), Dao Bo Hon, Hang Bo Nau Cave	North-eastern Vietnam	Golovatch et al. 2009a
23	*E. cambodiensis* sp. nov.	Cambodia, Kampot Province, Banteay Meas District, Prasat Phnom Totong	Southern Cambodia	This study
24	*E. communicans* Golovatch et al., 2009	Vanuatu, Espirito Santo, Malo Island, Avorani	Melanesia	Golovatch et al. 2009a
25	*E. curticornis* Golovatch et al., 2009	Vietnam, Nghê An Province, Anh Son: Hoi Son, Hang Lung Bo Cave	North-central Vietnam	Golovatch et al. 2009b
26	*E. demangei* Silvestri, 1910	Vietnam, Hanam Province, Phu-Ly	Northern Vietnam	Silvestri 1910; Enghoff et al. 2004; Golovatch et al. 2009a, 2009b
27	*E. digitatus* Liu & Tian, 2013	China, Guangdong Province, Qingyuan City, Jintan Town, Cave Mi Dong	Southern China	Liu and Tian 2013; Golovatch et al. 2015
28	*E. distinctus* Golovatch et al., 2009	China, Guangxi Province, Fushui, Bapen, Cave 4	Southern China	Golovatch et al. 2009b, 2015
29	*E. elegans* (Miyosi, 1956)	Japan, Enosima, Mizonokuti, Aoga-Sima (Idzu-Inseln) (Aogashima island)	Eastern Japan	Miyosi 1956; Golovatch et al. 2009a, 2010
30	*E. filisetiger* Golovatch et al., 2009	Vietnam, Thanh Hoa Province, Th anh Son: Lang Kho Muong, Hang Doi Cave	Northern Vietnam	Golovatch et al. 2009b
31	*E. gremialis* (Hoffman, 1982)	Thailand, Chiang Mai Province, Chiang Dao District, Chiang Dao caves	Northern Thailand	Hoffman 1982a; Golovatch et al. 2009a, 2009b
32	*E. griseus* Golovatch et al., 2009	Vietnam, Kien Giang Province, Kien Luong: Hon Chong, Nui Hon Chong, outside Cave 2 near Hang Hai Côt	Southern Vietnam	Golovatch et al. 2009b
33	*E. incisus* Golovatch et al., 2009	China, Guizhou Province, Qianxi County, Hong Lin Village, Tiao Shuz Dong Cave	South-western China	Golovatch et al. 2009a, 2015
34	*E. jianjia* Liu & Wynne, 2019	China, Guangxi Zhuang Autonomous Region, Yangshuo County, Guanshan No. 4 Cave	Southern China	Liu and Wynne 2019
35	*E. latellai* Golovatch et al., 2015	China, Guizhou Province, Zhen Feng County, Bei Pan Jiang Town, Cave Shui Chi Dong (Water Pool Cave)	South-western China	Golovatch et al. 2015
36	*E. latus* Golovatch et al., 2009	China, Guangxi Province, Yachang Nature Reserve, Yan Wu Dong Cave	Southern China	Golovatch et al. 2009a, 2015
37	*E. monodentus* (Zhang *in* Zhang & Wang, 1993)	China, Yunnan Province, Mengla County, Caiyun Cave	South-western China	Zhang and Wang 1993; Golovatch et al. 2009a, 2009b, 2015
38	*E. multilobatus* Golovatch et al., 2009	Laos, Luang Prabang Province, Nong Kiaw: Tham Pha Kouang, Cave B	Northern Laos	Golovatch et al. 2009b
39	*E. nadan* Golovatch et al., 2016	Laos, Khammouane Province, Ban Nadan, Cave Tham Nadan	Central Laos	Golovatch et al. 2016
40	*E. obliteratus* Golovatch et al., 2015	China, Guizhou Province, Guanling County, Huajiang Town, Cave Huashiban Dong (Slippery Cave)	South-western China	Golovatch et al. 2015
41	*E. parvus* Liu & Wesener, 2017	Laos, Huaphan Province, Cave Tham Nam Long	Eastern Laos	Liu et al. 2017
42	*E. peculiaris* (Murakami, 1966)	Japan, Shikoku, Ehime Prefecture, Niihama, Oshima	Southwestern Japan	Murakami 1966; Golovatch et al. 2009a, 2010
43	*E. planatus* Liu & Tian, 2013	China, Guangxi Zhuang Autonomous Region, Hechi City, Liujia Town, Cave Zhenzhuyan	Southern China	Liu and Tian 2013; Golovatch et al. 2015
44	*E. reductus* Golovatch et al., 2009	Indonesia, Sulawesi Selatan, kab. Maros: Samanggi, Gua Saripa Cave	Eastern Indonesia: Sulawesi	Golovatch et al. 2009b
45	*E. regularis* Golovatch et al., 2009	Vietnam, Lao Cai Province, Sa Pa, Hang Ta Phin Cave	North-western Vietnam	Golovatch et al. 2009b
46	*E. silvaticus* (Haga, 1968)	Japan, Kyushu Island, Fukuoka Prefecture, Tagawa City, Hojoo-machi, Gooya	South-western Japan	Haga 1968; Golovatch et al. 2010
47	*E. similis* Golovatch et al., 2009	China, Guangxi Province, Mulun Nature Reserve, Gui Dong 2 Cave	Southern China	Golovatch et al. 2009a, 2015; Liu and Tian 2013
48	*E. simplex* Liu & Tian, 2013	China, Jiangxi Province, Fenyi County, Cave Taoyuan Dong	East-central China	Liu and Tian 2013; Golovatch et al. 2015
49	*E. sketi* Golovatch et al., 2015	China, Hunan Province, Longshan County, Huaoyan, Cave Feihu Dong	Central China	Golovatch et al. 2015
50	*E. spinatus* Liu & Tian, 2013	China, Hunan Province, Sidu Town, Sidu Caves	Central China	Liu and Tian 2013; Golovatch et al. 2015
51	*E. taiwanensis* Golovatch et al., 2010	Taiwan, Taipei City, Wenshan District, Chih-Nan Temple	All parts of Taiwan	Golovatch et al. 2010, 2011
52	*E. tenuis* Golovatch et al., 2015	China, Guizhou Province, Guanling County, Yong Ning Town, Cave Yun Dong (Cloud Cave)	South-western China	Golovatch et al. 2015
53	*E. triangularis* Golovatch et al., 2015	China, Sichuan Province, Beichuan County, Cave Yuan Dong	South-western China	Golovatch et al. 2015
54	*E. troglobius* Golovatch et al., 2015	China, Guizhou Province, Kaiyang, Cave Xianyan Dong	South-western China	Golovatch et al. 2015
55	*E. trontelji* Golovatch et al., 2015	China, Guizhou Province, Libo County, Libo, Cave Feng Dong	South-western China	Golovatch et al. 2015

###### Updated diagnosis of the genus *Eutrichodesmus* Silvestri, 1910.

Golovatch et al. (2009a, 2009b) provided a complete diagnosis of the genus, as well as the main structural details of all genera in the family Haplodesmidae. It is therefore relatively easy to provide a morphological overview of *Eutrichodesmus*. However, as the genus shares some characters with certain confamilial genera, i.e. *Cylindrodesmus* Pocock, 1889; *Doratodesmus* Cook *in* Cook and Collin, 1985; and *Helodesmus* Cook, 1896, a refined diagnosis seems to be warranted. The more so as, since 2009, 18 further species of *Eutrichodesmus* have been described, adding a number of morphological traits across the genus (Makhan 2010; Golovatch et al. 2010, 2015, 2016; Liu et al. 2017; Liu and Wynne 2019). The amended diagnosis of *Eutrichodesmus* is chiefly based on that by Golovatch et al. (2009a, 2009b).

The genus *Eutrichodesmus* differs from all other Haplodesmidae by showing the following combination of characters. Body small (ca. 3.5–14 mm in length), with 19–20 rings; usually “doratodesmid” (= capable of volvation); conglobation usually complete, but sometimes incomplete. Tegument: collum and metaterga usually microgranulate and microvillose; prozonae often alveolate. Metatergawith or without mid-dorsal projections (outgrowths); usually with two or three rows of conspicuous tubercles (seldom four or more), often arranged mixostictic (irregular in axial direction) or, sometimes, isostictic (regular in axial direction). Paraterga short or long, usually lobulate. Ozopores usually present on rings 5, 7, 9, 10, 12, 13, 15–19, rarely reduced or absent; with or without porosteles. Gonopod: Coxae often microgranulate; usually abundantly setose, sometimes with a distolateral outgrowth. Telopodite usually long and slender; basal half of telopodite (= prefemoral part) densely setose; often with a distofemoral process, conspicuous, located laterally on femorite, sometimes absent. Acropodite well-developed, conspicuous. Solenomere often completely fused to acropodite (solenomere = acropodite), rarely separated and forming a lobe. Seminal groove running on mesal side of prefemur, usually terminating at about halfway of acropodite to distal region; with or without hairpad.

#### Description of the new species

##### Eutrichodesmus cambodiensis

Taxon classificationAnimaliaPolydesmideaHaplodesmidae

Srisonchai & Panha sp. nov.

8155AF98-9B65-517A-B7D1-18DEC16174A0

http://zoobank.org/10A8DC52-D01C-4892-8B20-88D9F0EBB009

Figures 1 , 2 , 3 , 4 , 5W , 7T , 9 , 10

###### Material examined.

***Holotype*** male (CUMZ–hpd0001), CAMBODIA, Kampot Province, Dang Tong District, near Wat Phnom Small, limestone hills, 10°42'12"N, 104°31'30"E, ca. 47 m AMSL, leg. C. Sutcharit, W. Siriwut, E. Jerutthitikul, P. Trach, S. Chuoy & R. Srisonchai (locatily no. C041), 16 September 2019. ***Paratypes*.** Twenty-three males, fifteen females (CUMZ–hpd0002), same data as holotype. Six males and six females (CUMZ–hpd0002) CAMBODIA, Kampot Province, Banteay Meas District, Prasat Phnom Totong, 10°41'49"N, 104°31'23"E, ca. 31 m AMSL, leg. C. Sutcharit, W. Siriwut, E. Jerutthitikul, P. Trach, S. Chuoy & R. Srisonchai (locatily no. C042), 16 September 2019. One male, one female (CIFI), same data as holotype. One male (ZRC_ENT00014160), one female (ZRC_ENT00014161), same data as holotype. ***Further specimens, non-types*.** Two broken males, two broken females, one male without gonopods, eight juveniles, two males and two females prepared for DNA extraction (CUMZ–hpd0002), same data as for holotype.

###### Etymology.

The specific epithet reflects the name of the country “*Cambodia*” where all specimens were collected and to which the new species appears to be endemic; adjective.

###### Diagnosis.

Body with incomplete volvation; metaterga with three transverse rows of regular and round tubercles, but no mid-dorsal projection (outgrowth) on metaterga; distofemoral process on gonopod telopodite very short, inconspicuous. Similar in all these characters to *E. griseus* Golovatch et al., 2009, but differs in having (1) live specimens and freshly preserved material pale greyish-brown or pale brown in colour; (2) the limbus crenulate, but not spinulate, crenulations being slightly longer than broad; (3) the acropodite curved and long, unciform, attenuated near tip; with a free solenomere starting from about midway; and (4) the solenomere digitiform, papillate, without hairpad.

###### Description.

Body length 5–7 mm (male) or 6–8 mm (female); width of mid-body metazonae ca. 0.9 mm (male) or ca. 1.2 mm (female). In width, head < collum < 2 = 3 < 4 < 5–17, thereafter body gradually tapering towards telson. Females apparently longer and larger than males.

**Figure 1.**
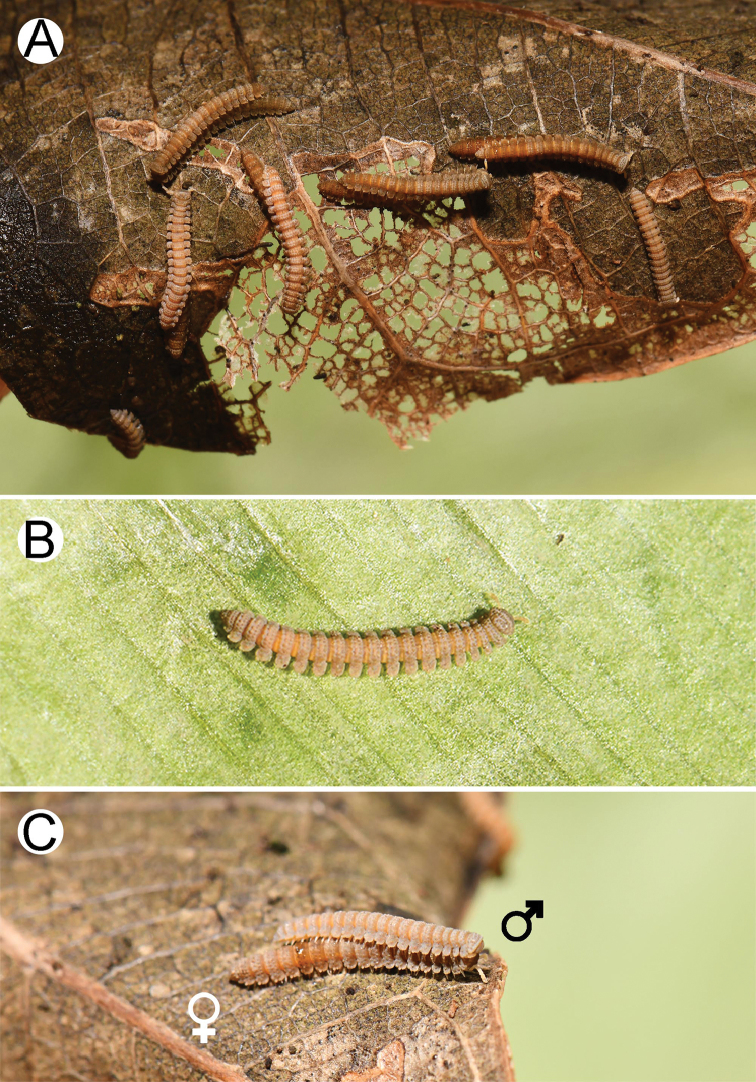
Photographs of live *Eutrichodesmus cambodiensis* sp. nov., paratypes (CUMZ–hapld00002) **A** pairs of mating couples **B** male and **C** mating couple. Not to scale.

***Colour*** (Fig. 1). Live specimens pallid greyish-brown or brown: head grey; antennae pale brown; collum, metaterga and paraterga greyish-brown; surface below paraterga, prozonae, sterna and legs brown. Specimens in alcohol after six months of presevration nearly the same in colour as in life.

**Figure 2.**
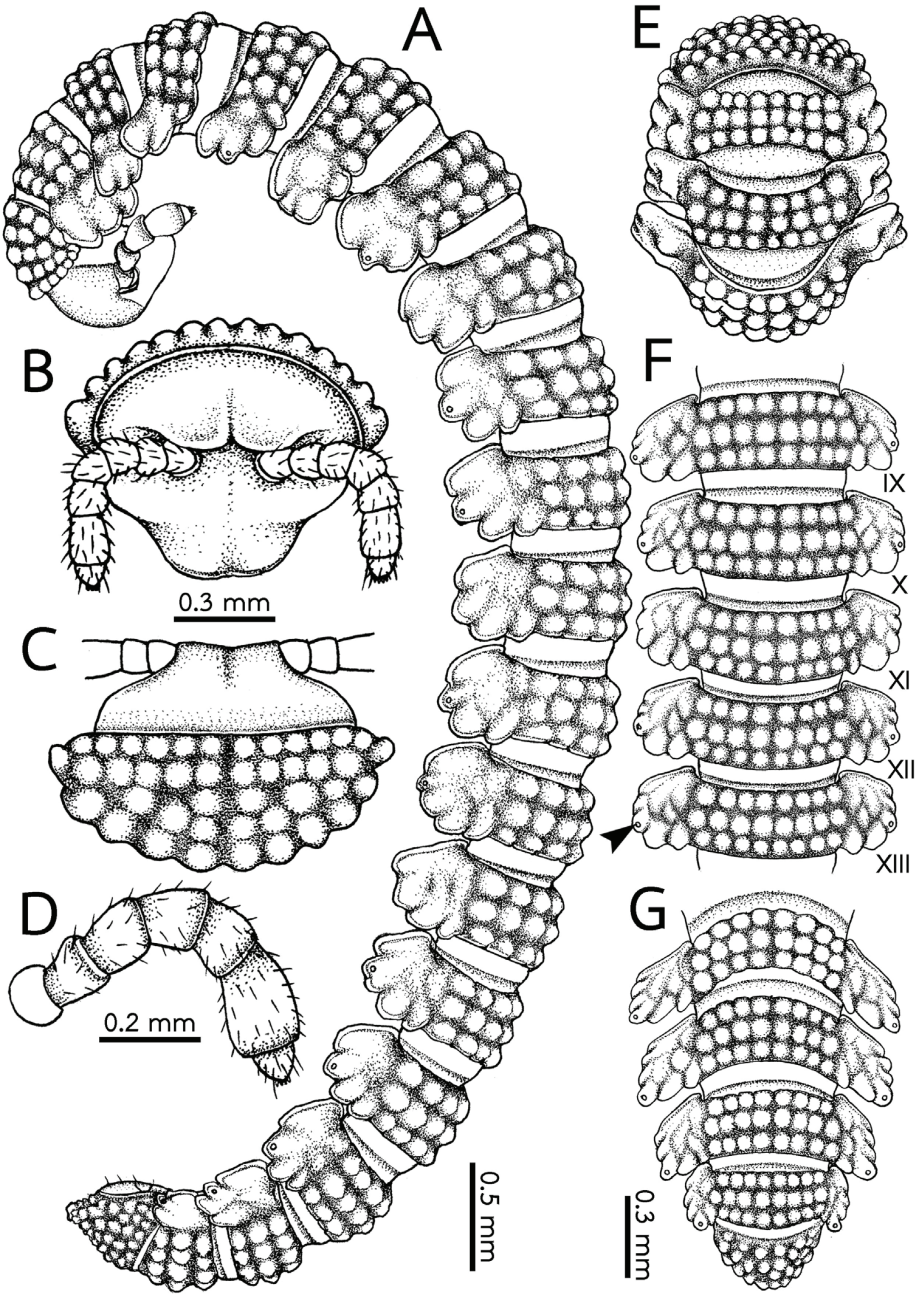
*Eutrichodesmus cambodiensis* sp. nov., male paratype (CUMZ–hpd0002) **A** whole body part **B** head and antenna **C** head and collum **D** antenna **E** anterior body part **F** body rings 9–13 (arrowhead points to ozopore) **G** posteriormost body rings and telson **A** lateral view **B** anterior view **C, E–G** dorsal view. Scale bars: 0.3 mm (**B, C**), 0.3 mm (**E–G**).

***Body*** (Fig. 2A). General appearance as in Fig. 2A. Body long and slender. Adults with 20 rings. Volvation incomplete because of a slender body and short paraterga.

***Head*** (Fig. 2B, C). Slightly transverse, wider than high, densely pilose, not covered with collum from above. Vertex microvillose and microgranulate. A pair of small, poorly separated, paramedian knobs above antennal sockets. Isthmus between antennae ca. 1.3 times as wide as diameter of antennal socket. Epicranial suture deep and conspicuous. Labrum and genae sparsely setose.

***Antennae*** (Fig. 2B, D). Short and stout, clavate, densely setose, setae being long.

Antennomere 6 longest, with a group of bacilliform sensilla located inside a shallow distolateral pit near tip of each of antennomeres 5 and 6. Antennomere 8 with four sensory cones apically.

**Figure 3.**
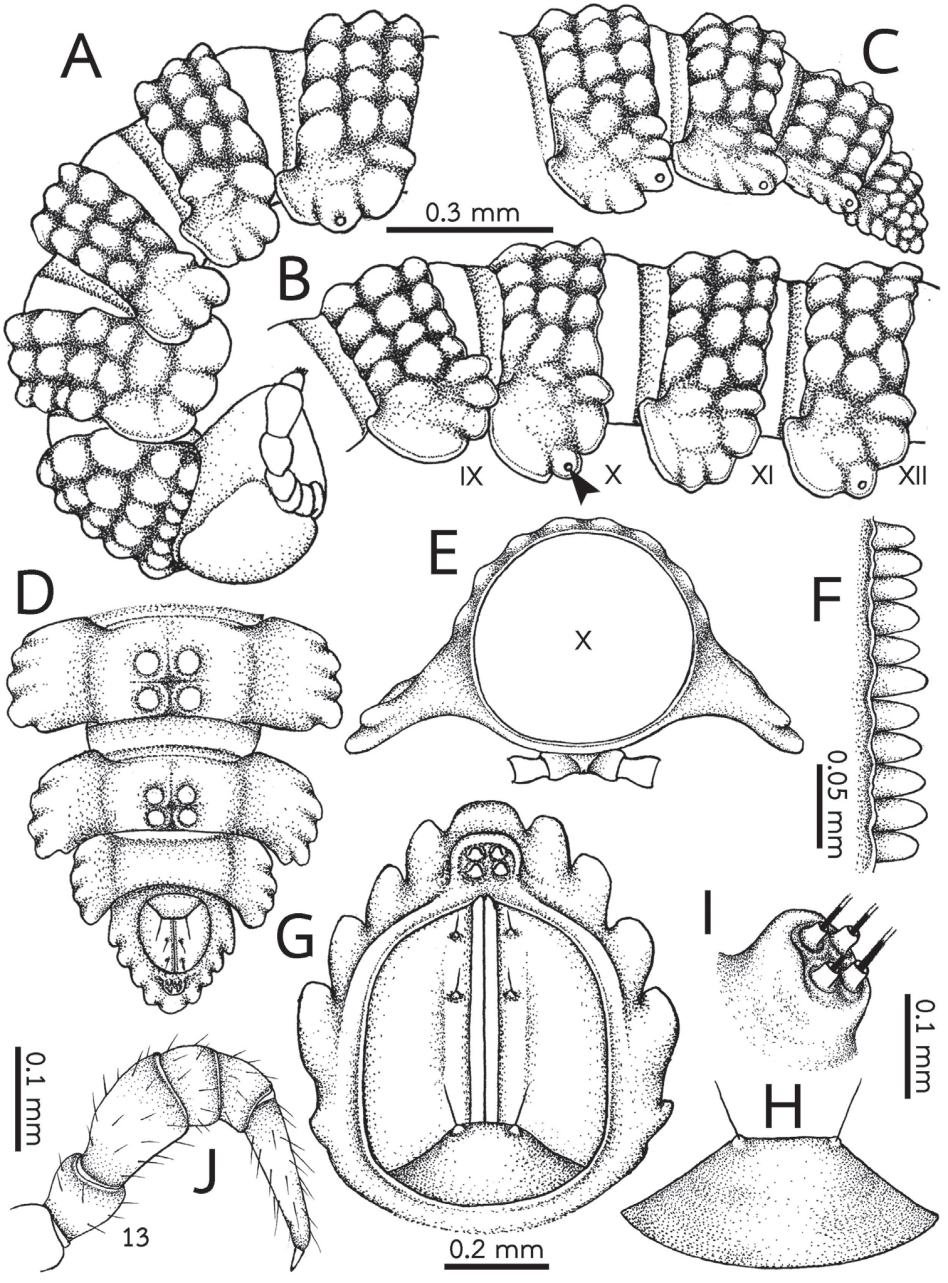
*Eutrichodesmus cambodiensis* sp. nov., male paratype (CUMZ–hpd0002) **A** anterior body part **B** body rings 9–12 (arrowhead points to ozopore) **C, D** posteriormost body rings and telson, **E** mid-body ring **F** limbus of body ring 10 **G** last ring and telson **H** hypoproct **I** two pairs of apical setae (spinnerets) **J** leg 13 **A–C, I** lateral view **D, G, H** ventral view. Scale bars: 0.5 mm (**A–E**), 0.1 mm (**H, I**).

***Collum*** (Figs 2A, C, E; 3A). Large, nearly semi-circular, a little broader than head, with regular and rounded tubercles arranged in five transverse rows: usually 7+7 tubercles in anterior (first) row, followed by 4+4, 1(0)+1(0), 3+3 and 4+4 tubercles in rows 2–5, respectively. Anterior margin truncate, slightly elevated, resembling that in *E. griseus* or some species of Pyrgodesmidae. Posterior margin round. Lateral margin narrow, directed laterad.

***Tegument***. Overall quite dull, some specimens encrusted with dirt. Head mostly microgranulate (labrum and clypeus smooth). Collum microvillose. Prozonae finely alveolate.

Suture between pro- and metazonae quite shallow and broad, more strongly alveolate and microgranulate than prozonae. Metaterga, paraterga, surface below paraterga, sterna, epiproct and hypoproct microgranulate and microvillose. Legs smooth.

***Metaterga*** (Figs 2A, E–G; 3A–C). Metaterga2–19 each with three transverse rows of undiffentiated tubercles, pattern isostictic (arrangement regular in axial position). Anterior, intermediate and posterior rows each with 4+4 tubercles; those in second row on body rings 5–19 larger than in other rows. Each tubercle anteriorly with a short, spatulate, bisegmented seta. Mid-dorsal (axial) line missing.

***Limbus*** (Fig. 3F). Crenulate, each lobulation being slightly longer than broad, tip round.

***Paraterga*** (Figs 2A, E–F; 3A–C, E; 5W). Broad, slightly sloping down. Tip round and directed ventrolaterad. Paraterga 2 enlarged, *in situ* more strongly sloping down than on other rings, with four or five conspicuous lobules. Paraterga 3 and 4 shorter, narrower than others; each with four conspicuous lobules. Paraterga 5–18 mostly with four lobules, some rings with five ones.

***Ozopores*.** Inconspicuous when seen in dorsal view. Pores small, oval in shape, lacking a porostele, opening laterally near rear margin of paraterga above it. Pore formula normal (5, 7, 9, 10, 12, 13, 15–19).

***Pleurosternal ridges***. Absent.

***Epiproct*** (Fig. 3D, G, I). Very short, flattened dorsoventrally; knob-like apically, with two pairs of inconspicuous setae (spinnerets), each spinneret located inside a tube-like structure, both dorsal and ventral spinnerets arranged inside a circular shallow depression.

***Paraproct*** (Fig. 3G). Normal, with two pairs of small setae.

***Hypoproct*** (Fig. 3G, H). Subtrapeziform, caudal margin truncate, with two small, inconspicuous, setiferous tubercles.

***Spiracle***. Simple, located above anterior and slightly before posterior legs.

***Legs*** (Fig. 3J). Quite short and stout, in situ almost reaching the tip of paraterga. Relative length of podomeres: tarsus > femur > (prefemur ≥ coxa) = postfemur = tibia > claw.

***Sterna*** (Fig. 3D). Narrow. Longitudinal depression between coxae in most body rings deep and narrow, only in ring 7 quite deep and wide for accommodating the shafts of gonopods. Transverse depression deep and wide.

***Gonopod aperture***. Very large, transversely ovoid, subequal to width of prozonite.

**Figure 4.**
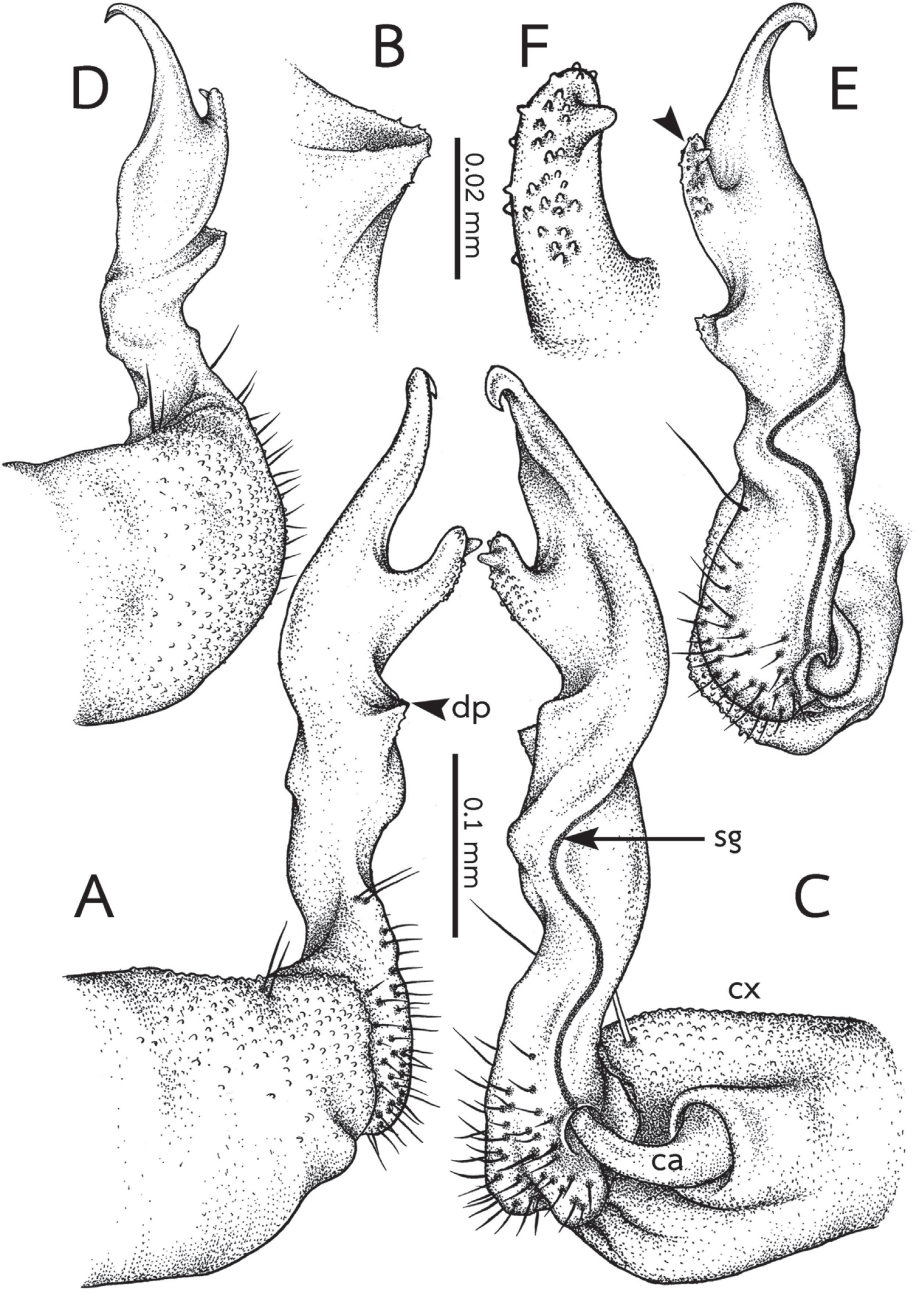
*Eutrichodesmus cambodiensis* sp. nov., right gonopod, male paratype (CUMZ–hpd0002) **A** lateral view (dp = distofemoral process) **B** distofemoral process **C** mesal view (cx = coxa, cn = cannula, sg = seminal groove) **D** dorsal view **E** ventral view (arrowhead points to solenomere) **F** digitiform solenomere. Scale bars: 0.1 mm (**A, C–E**), 0.02 mm (**B, F**).

***Gonopods*** (Figs 4; 7T). Shafts when retracted reaching the anterior part of sternum 7 (base of legs 8). Coxa (cx) large and stout, subquadrate, microgranulate, with a few short setae distolaterally. Cannula (cn) simple, conspicuous, curved and slender, swollen at base, inserted into a small depression at base of telopodite on posteromedial side. Telopodite suberect; basal half (= prefemoral part) nearly straight; distal half curved. Distofemoral process (dp) very short, located at about midway of telopodite, triangular, dentate. Acropodite conspicuous, with neither a lobe nor a process, distally slightly attenuated and forming a hook-like tip, directed and curved mesad. Solenomere partially separated from acropodite, conspicuous, digitiform, papillate, originating at ca. 3/4 height of telopodite beyond distofemoral process; rather short, tip *in situ* directed anteriad, apically with a large papilla which is more conspicuous than other papillae. Seminal groove (sg) conspicuous, thick, running entirely on mesal surface of telopodite, terminating without hairpad by opening on the large papilla of solonomere.

###### Remarks.

Although the genital characters of females have not been used for taxonomic purposes in the present study, all females were examined. In all cases, the female non-genital characters were found similar to those found in males. The only difference which can be clearly seen using both live and preserved material is that females are apparently broader and longer than males (Fig. 1C).

The general colouration does not show any variability and the paratypes do not differ significantly from the holotype. Across the type series of the new species, there was little intrapopulational variation in the number of tubercles on the collum and of lobes on the paraterga: an intermediate row (third or middle row) of the collum usually showed 1+1 tubercles, only sometimes 0+1 or 1+0 tubercles; paraterga of most specimens usually had four conspicuous lobes, only sometimes five. However, all these variations in most of the non-gonopodal characters were minor, neither significant nor consistent enough to be useful for taxonomic purposes, at least in the species under consideration. Little can be said about interpopulational variation in the new species because no variation has been noted between the two examined populations and no other specimens living at and around these two locations have been found.

Notably, *E. cambodiensis* sp. nov. shows a slightly elevated anterior margin of the collum (Figs 2A, 3A). As this can easily be seen also in *E. griseus*, it is consistent with what Golovatch et al. (2009b) found. Currently, only these two *Eutrichodesmus* species have the collum elevated in the anterior part, this strongly resembling the typical condition in the micropolydesmoid family Pyrgodesmidae.

The new species has the same characters as found in a bunch of congeners and shares the combination: adults with 20 body rings; body with incomplete volvation; metaterga without mid-dorsal projections, with three transverse rows of tubercles; and gonopod telopodite with a distofemoral process. All above characters are present in *E. basalis* Golovatch et al., 2009; *E. curticornis* Golovatch et al., 2009; *E. demangei* Silvestri, 1910; *E. filisetiger* Golovatch et al., 2009; *E. gremialis* (Hoffman, 1982); *E. griseus*, *E. multilobatus* Golovatch et al., 2009; *E. nadan*Golovatch et al., 2016; *E. parvus* Liu & Wesener, 2017 and *E. regularis* Golovatch et al., 2009 (see also Key and Table 2). Even though some traits have been observed, shared, especially in the gonopodal telopodite, between-species differences are always marked. With respect to the most relevant feature which lies in certain details of gonopodal structure, *E. cambodiensis* sp. nov. seems to be morphologically more similar to *E. griseus* than to any other congener, in particular in having a very short distofemoral process and the solenomere partly separated from the acropodite by forming a conspicuous lobe.

**Table 2.** Morphological comparison of some congeners with *E. cambodiensis* sp. nov. and distribution.

Characters	*E. cambodiensis* sp. nov.	*E. curticornis* Golovatch et al., 2009	*E. filisetiger* Golovatch et al., 2009	*E. griseus* Golovatch et al., 2009	*E. nadan* Golovatch et al., 2016	*E. parvus* Liu & Wesener, 2017	*E. regularis* Golovatch et al., 2009
**Colour of living specimens**	greyish-brown or pallid brown	uniformly pallid (probably greyish-brown)	uniformly pallid (probably light brown)	grey to blackish	uniformly light creamy-brown	uniformly light yellow-brown (probably light brown)	uniformly pallid (probably light brown)
**Rows of tubercles on collum**	5, regular tubercles	~5, regular tubercles	~5, irregular tubercles	5, regular tubercles	4–5?, very flat & round tubercles	5, round tubercles	5, regular tubercles
**Rows of tubercles on metaterga**	3 (isostictic), with inconspicuous setae	3 (mixostictic), with inconspicuous setae	3 (mixostictic), with filiform setae	3 (isostictic), with inconspicuous setae	3 (mixostictic), with inconspicuous setae	3 (mixostictic), with inconspicuous setae	3 (isostictic), with inconspicuous setae
**Limbus**	with round lobes, longer than broad (crenulate)	crenulate	spiculate	spinulate	microcrenulate	microcrenulate	crenulate
**Paraterga**	very high	low	very high	very high	very low	moderately high	moderately high
**Distofemoral process (dp)**	short, inconspicuous, triangular, dentate	extremely long, denticulate	very short, inconspicuous, triangular	short, inconspicuous, triangular	very long, denticulate	extremely, denticulate	long, digitiform, papillate
**Acropodite**	very long, without lobe	long, with a small lobe (tooth-like)	very long, with a bifid lobe	long, without lobe	Quite short, with a small lobe subapically	very long, subapically with a tooth & a lobe	with 2 lobes (small denticles)
**Solenomere**	long lobe, digitiform with papillate (no hairpad)	completely fused with acropodite (no hairpad)	completely fused with acropodite, with hairpad	long lobe, digitiform, with hairpad	completely fused with acropodite, with conspicuous hairy pulvillus	completely fused with acropodite (no hairpad)	lamelliform, fused with acropodite, with pilose-spinulate pulvillus
**Distribution**	Southern Cambodia	North-central Vietnam	Northern Vietnam	Southern Vietnam	Central Laos	Eastern Laos	North-western Vietnam

**Figure 5.**
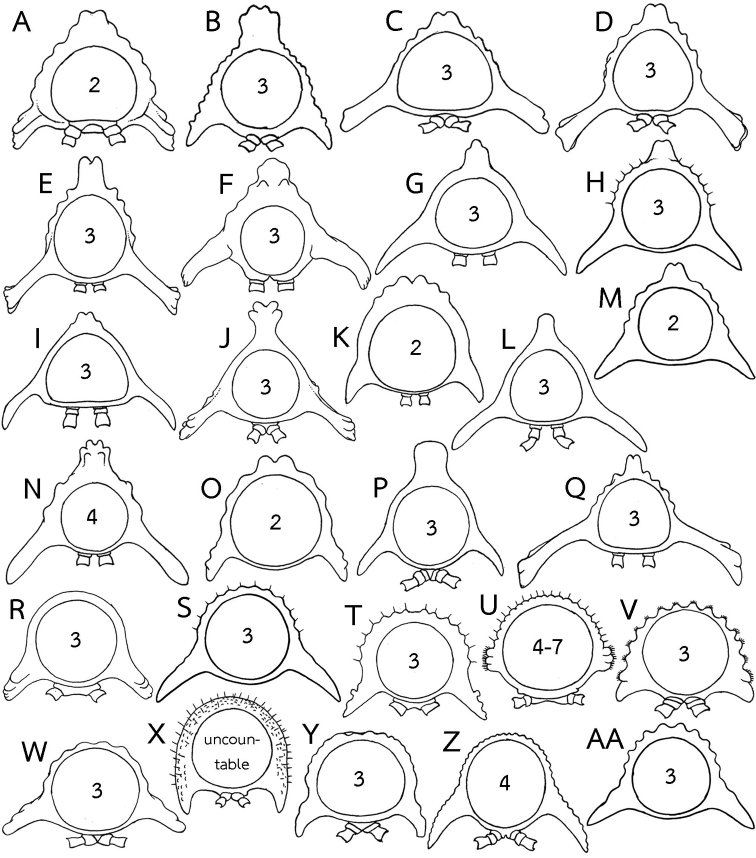
Body ring (posterior view) in several *Eutrichodesmus* species, number inside ring indicated rows of tubercles on metaterga. **A–Q** Species with mid-dorsal projections on metaterga. **A***E. anisodentus***B***E. aster***C***E. asteroides***D***E. astriproximus***E***E. astrisimilis***F***E. cavernicola***G***E. deporatus***H***E. dorsiangulatus***I***E. lipsae***J***E. macclurei***K***E. nodulosus***L***E. paraster***M***E. pectinatidentis***N***E. reclinatus***O***E. soesilae***P***E. steineri* and **Q***E. subasteroides***R–AA** Species without mid-dorsal projections on metaterga **R***E. apicalis***S***E. arcicollaris***T***E. armatocaudatus***U***E. armatus***V***E. basalis***W***E. cambodiensis* sp. nov. **X***E. communicans***Y***E. curticornis***Z***E. digitatus* and **AA***E. distinctus*. Not to scale. Figures modified from **A, M** Zhang (1995) **B, C, Y, AA**Golovatch et al. (2009b)**D, E, Q**Golovatch et al. (2016)**F**Sinclair (1901)**E, L, P**Liu and Wesener (2017)**H, S** Zhang in Zhang and Wang (1993)**I, R**Golovatch et al. (2015)**J, N** Hoffman (1977) **K**Verhoeff (1939)**O**Makhan (2010)**T, V, X**, Golovatch et al. (2009a)**U**Miyosi (1951) and **Z**Liu and Tian (2013).

###### Distribution and habitat.

It is worth noting that the new species was found only at the two sites. Surveys of other limestone and sandstone habitats surrounding the type locality (Kampong Trach) over a period of approximately two years have revealed no further specimens (Fig. 10). In showing a distribution of only two locations in a small and isolated limestone area, the new species can be suggested as being not only endemic to Cambodia, but also indigenous in Kampong Trach.

All specimens of the new species were hand-collected and found walking on humid rock walls of limestone caves (Fig. 9A). The vast majority of millipedes were seen crawling on humid rocks, whereas only a minor part was found slowly walking on vegetation, shaded holes and rock crevices during the daytime (Fig. 9B, C, E). It is important to note that specimens were commonly found under herb patches in a slightly shaded moist rock where the plant genus *Epithema* Blume, 1826 (family Gesneriaceae) created a mass of roots and thin litter layer on the soil in the hole (Fig. 9C). This is probably a particular microhabitat for *E. cambodiensis* sp. nov. Furthermore, we noted a co-occurrence between *E. cambodiensis* sp. nov. and the abundant *Hypselostoma cambodjense* Benthem Jutting, 1962, a microsnail (Fig. 9D), within a portion of the moist rock walls, as well as in rock crevices, but without being sympatric with other millipedes in the same microhabitats.

The habitat preferred by the new species clearly appears to be limestone, especially near caves, although all specimens were found outside the caves, near the entrance zones. No material was collected at twilight, transition or deep zones inside the cave [for a characterisation of the zonal environment in caves, see Liu and Wynne (2019)]. Many of the small holes/caves at the type locality where *E. cambodiensis* occurs are highly humid and have diminished light, owing to the shade from large trees in the area.

A large concern would be the ongoing habitat destruction very close to the type locality, where a cement factory is located on the opposite side of the mountain. Many outcrops in the area appear to have been quarried and it seems plausible that the existence of the type locality would be threatened in the near future.

###### Observation of mating behaviour.

Interestingly, all specimens of the new species collected around moist organic material and plants near the caves were pairs of several mating couples (Figs 1C, 9E). No single males or females were found separately. One presumption would be that individual millipedes were perhaps hidden in rock crevices during the daytime. The pairs of the new species mated during the rainy season when the rate of annual rainfall amount is quite high, which may imply the peak in mating occurring around September. The initial observations of the courtship were made by separating seven pairs into individual airflow plastic vials without human disturbance and we found that males appeared to initiate copulation by approaching the female from behind and then slightly reaching to the head region. The male took at least five hours grasping onto a female by its legs before it entwined and finally inserted its gonopod shaft into the female’s vulva.

### Notes on species groups in *Eutrichodesmus*

Table 3 summarises some significant characters across *Eutrichodesmus* species.

**Table 3.** Species groups of *Eutrichodesmus* Silvestri, 1910 and their main morphological characters.

Group name	Species	Morphological characters				Distribution
		Body volvation	Rows of tubercles on metaterga	Mid-dorsal projection on metaterga	Distofemoral process on telopodite	
***peculiaris*-group** (7 spp.) (established by Golovatch et al. 2010)	*E. anisodentus*	complete	2	present	absent	Japan, Taiwan, and China
	*E. nodulosus*			present		
	*E. pectinatidentis*			present		
	*E. peculiaris*			absent		
	*E. silvaticus*			present		
	*E. soesilae*			present		
	*E. taiwanensis*			absent		
***demangei*-group** (46 spp.)	*E. apicalis*, *E. arcicollaris*, *E. armatocaudatus*, *E. armatus*, *E. aster*, *E. asteroides*, *E. astriproximus*, *E. astrisimilis*, *E. basalis*, *E. cambodiensis* sp. nov., *E. cavernicola*, *E. curticornis*, *E. demangei*, *E. deporatus*, *E. digitatus*, *E. distinctus*, *E. dorsiangulatus*, *E. elegans*, *E. filisetiger*, *E. gremialis*, *E. griseus*, *E. incisus*, *E. jianjia*, *E. latellai*, *E. latus*, *E. lipsae*, *E. macclurei*, *E. monodentus*, *E. multilobatus*, *E. nadan*, *E. obliteratus*, *E. paraster*, *E. parvus*, *E. planatus*, *E. reclinatus*, *E. regularis*, *E. similis*, *E. simplex*, *E. sketi*, *E. spinatus*, *E. steineri*, *E. subasteroides*, *E. tenuis*, *E. triangularis*, *E. troglobius*, *E. trontelji*	complete/incomplete	mostly 3	present (17 spp.)/	present	Japan
			(rarely 4 or more, in *E. armatus* and *E. digitatus*)	absent (29 spp.)	(absent in *E. astriproximus*)	China and Mainland Southeast Asia
Ungrouped (2 spp.)	*E. communicans*	complete	numerous setae	absent	present (broad lobe)	Vanuatu
	*E. reductus*	incomplete	(no tubercle)		present (broad lobe)	Indonesia
			numerous setae			
			(no tubercle)			

The genus *Eutrichodesmus* was recently revised by Golovatch et al. (2009a, 2009b), who also refined the family and its generic classification, where many remarkable species were also described. Later, Golovatch et al. (2010) reported some sharable characters that can be used for more clearly delimiting species groups. The first and until now only species group, named the “*peculiaris*-group”, was proposed by Golovatch et al. (2010) and it currently encompasses seven species, viz; *E. anisodentus*, *E. nodulosus*, *E. pectinatidentis*, *E. peculiaris*, *E. silvaticus*, *E. soesilae* and *E. taiwanensis*, all sharing two rows of tubercles on the metaterga, having a broad and flattened epiproct, lacking a distofemoral process and with complete body volvation. Not only do these morphological traits strongly support this group, but their distribution is also likely to be coherent since most of the species inhabit the same region (southern part of Japan, Taiwan and mainland China).

**Figure 6.**
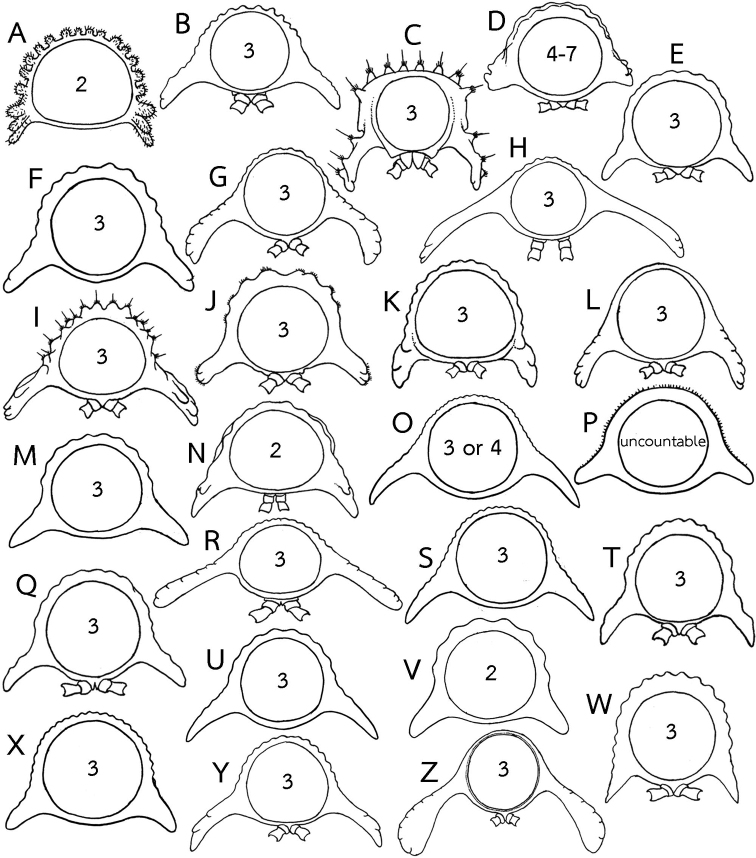
Body ring (posterior view) in several *Eutrichodesmus* species, number inside ring indicates rows of tubercles on metaterga. All species without mid-dorsal projections on metaterga **A***E. elegans***B***E. filisetiger***C***E. gremialis***D***E. griseus***E***E. incisus***F***E. jianjia***G***E. latellai***H***E. latus***I***E. monodentus***J***E. multilobatus***K***E. nadan***L***E. obliteratus***M***E. parvus***N***E. peculiaris***O***E. planatus***P***E. reductus***Q***E. regularis***R***E. similis***S***E. simplex***T***E. sketi***U***E. spinatus***V***E. taiwanensis***W***E. tenuis***X***E. triangularis***Y***E. troglobius* and **Z***E. trontelji*. Not to scale. Figures modified from **A**Miyosi (1956)**B, D, J, P, Q**Golovatch et al. (2009b)**C** Hoffman (1982) **E, H, R**Golovatch et al. (2009a)**F**Liu and Wynne (2019)**G, L, T, W, X, Y, Z**Golovatch et al. (2015)**I** Zhang *in*Zhang and Wang (1993)**K**Golovatch et al. (2016)**M**Liu and Wesener (2017)**N**Murakami (1966)**O, S, U**Liu and Tian (2013) and **V**Golovatch et al. (2010).

**Figure 7.**
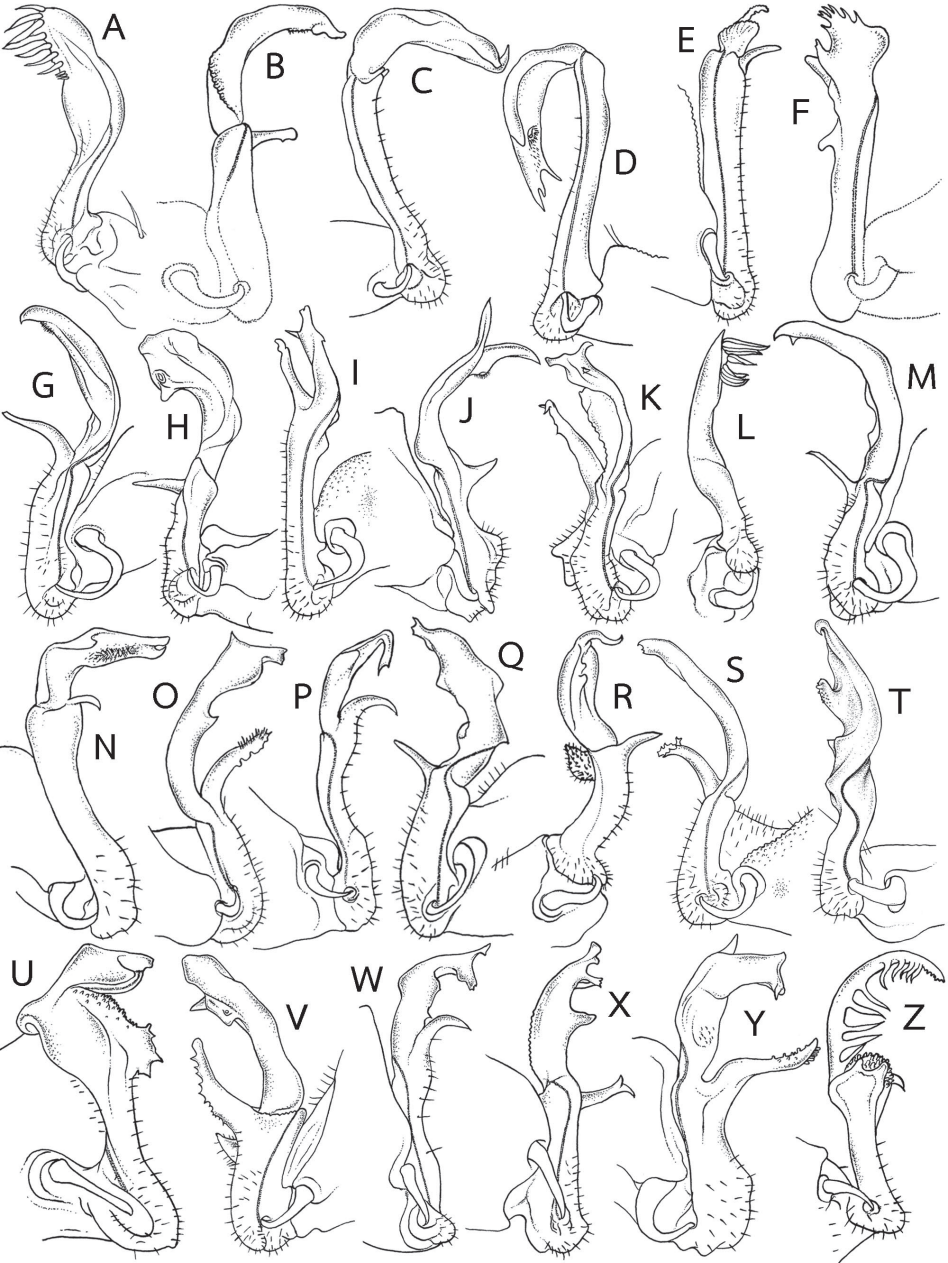
Gonopod outline (mesal view of left and right gonopod) in several *Eutrichodesmus* species. **A–N** Gonopods of species with mid-dorsal projections on metaterga **A***E. anisodentus***B***E. aster***C***E. asteroides***D***E. astriproximus***E***E. astrisimilis***F***E. cavernicola***G***E. deporatus***H***E. dorsiangulatus***I***E. lipsae***J***E. macclurei***K***E. paraster***L***E. pectinatidentis***M***E. steineri* and **N***E. subasteroides***O–Z** Gonopods of species without mid-dorsal projections on metaterga **O***E. apicalis***P***E. arcicollaris***Q***E. armatocaudatus***R***E. armatus***S***E. basalis***T***E. cambodiensis* sp. nov. **U***E. communicans***V***E. curticornis***W***E. demangei***X***E. digitatus***Y***E. distinctus* and **Z***E. elegans*. Not to scale. Figures modified from **A, L** Zhang (1995) **B, C, V, Y**Golovatch et al. (2009b)**D, E, N**Golovatch et al. (2016)**F**Sinclair (1901)**G, K, M**Liu and Wesener (2017)**H, P** Zhang in Zhang and Wang (1993)**I, O**Golovatch et al. (2015)**J** Hoffman (1977) **Q, S, U**Golovatch et al. (2009a)**R**Miyosi (1951)**W**Silvestri (1910)**X**Liu and Tian (2013) and **Z**Miyosi (1951).

**Figure 8.**
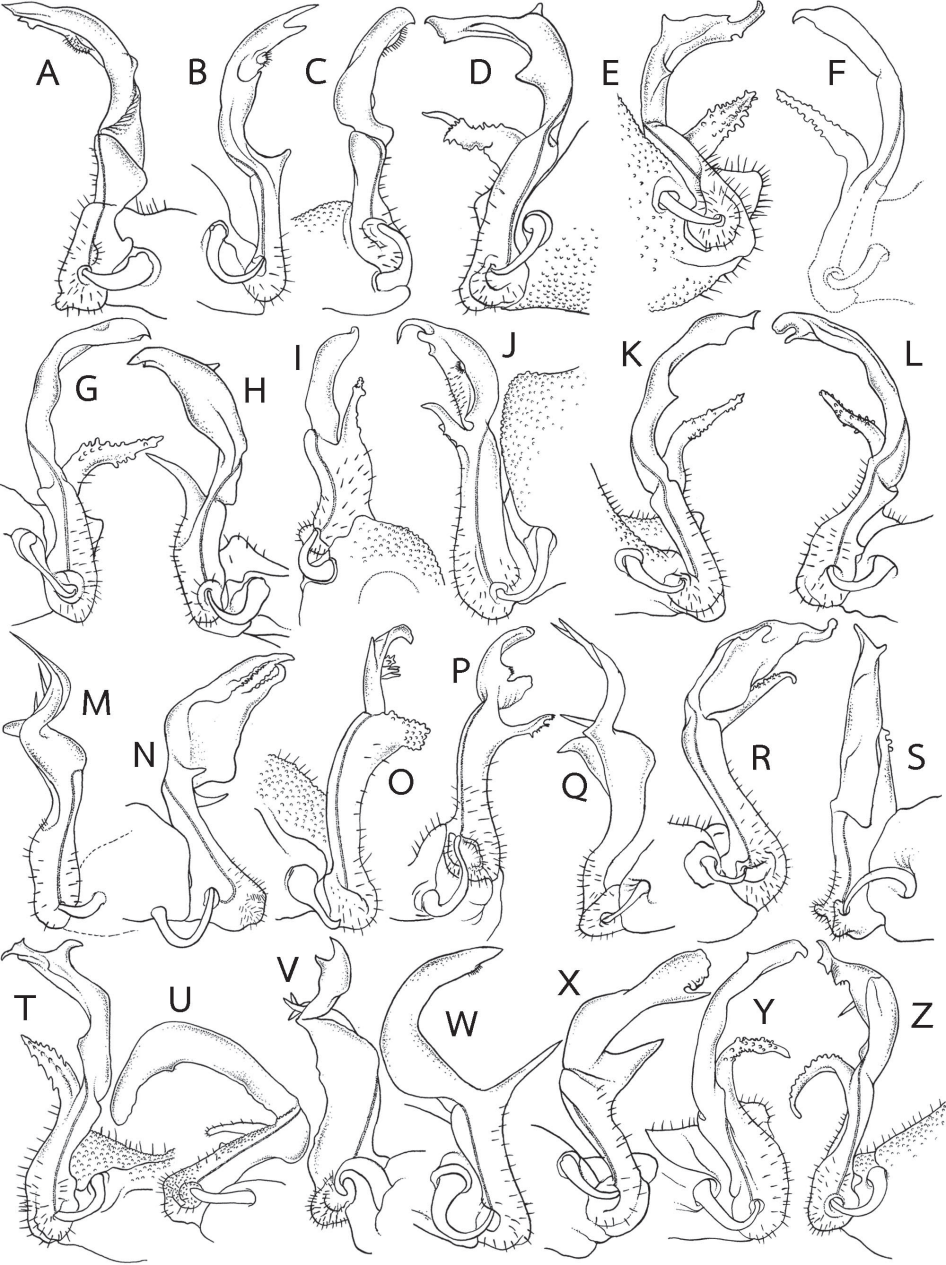
Gonopod outline (mesal view of left and right gonopod) in several *Eutrichodesmus* species. All species without mid-dorsal projections on metaterga. **A***E. filisetiger***B***E. gremialis***C***E. griseus***D***E. incisus***E***E. jianjia***F***E. latellai***G***E. latus***H***E. monodentus***I***E. multilobatus***J***E. nadan***K***E. obliteratus***L***E. parvus***M***E. peculiaris***N***E. planatus***O***E. reductus***P***E. regularis***Q***E. silvaticus***R***E. similis***S***E. simplex***T***E. sketi***U***E. spinatus***V***E. taiwanensis***W***E. tenuis***X***E. triangularis***Y***E. troglobius* and **Z***E. trontelji*. Not to scale. Figures modified from **D, G, R**Golovatch et al. (2009a)**A, C, I, O, P**Golovatch et al. (2009b)**B** Hoffman (1982) **E**Liu and Wynne (2019)**F, K, T, W, X, Y, Z**Golovatch et al. (2015)**H** Zhang in Zhang and Wang (1993)**J**Golovatch et al. (2016)**L**Liu and Wesener (2017)**M**Murakami (1966)**N, S, U**Liu and Tian (2013)**Q**Haga (1968) and **V**Golovatch et al. (2010).

The discovery of a new species from Cambodia not only represents the first record of the genus, but also of the entire family Haplodesmidae from that country. In this study, we do not only describe a new species, but we also update and compare the morphological characters of all currently known congeners, based on our scrutiny of all relevant original literature sources (Figs 5–8). The comparison, which relies mainly on details of gonopodal structure, body volvation patterns, the number and arrangement of the rows of tubercles and mid-dorsal projections on the metaterga, revealed an adequate delimitation for all 48 remaining species into another group for some coherent assemblages. We assemble 46 species into a second species group, here named the “*demangei*-group” and the remaining two species which are left ungrouped (see Table 3). Notably, these 46 species share some possibly related characters: metaterga usually with three rows of tubercles (except *E. armatus* and *E. digitatus* which have four or more rows); gonopod telopodite with a distofemoral process (absent from *E. astriproximus*). All constituent species of *Eutrichodesmus* are presented in Table 3.

The gonopod might be a reliable tool for natural species group delimitations and quite often the assignment of many haplodesmid groups has been based on these characters. Our discrimination has also found the gonopodal structure to be useful in providing several satisfactory characters for sorting out amongst *Eutrichodesmus* species. Figures 7, 8 clearly show that all species of the “*demangei*-group” show the same pattern of such gonopodal characters as the existence of a distofemoral process on the telopodite, combined with most species showing three rows of tubercles on the metaterga, while the mid-dorsal projection and body volvation seem to be variable across *Eutrichodesmus* (Figs 5, 6). In accordance, the distribution of the “*demangei*-group”, which all inhabit Japan, China and mainland Southeast Asia, corresponds to the morphological characters, although their distribution area is obviously larger. The other congeners, *E. nodulosus* and *E. reclinatus*, both lack gonopodal information yet, because they were originally described from females only. In spite of their gonopodal structure being unknown, their other morphological traits seem to fit in and serve to place these species in the “*demangei*-group” much more than to any other group.

Whereas the remaining 46 species agree in most respects with the definition of the “*demangei*-group” given above, there is a strong difference in the structural details of the gonopod, the presence of mid-dorsal projections and the number of the rows of tubercles on metaterga observed in two species, *E. communicans* and *E. reductus*. These can be assigned to neither the “*peculiaris*-group” nor the “*demangei*-group” due to the remarkable numerous setae without tubercles on the metaterga and the broad distofemoral process on the gonopod femorite, as well as their geographical distribution (*E. communicans* from Vanuatu and *E. reductus* from Indonesia) which quite clearly makes them separated from all other congeneric species. Thus, we leave *E. communicans* and *E. reductus* amongst ungrouped species as circumscribed above, since they fail to match the definition of the new or other previously-described species groups (see also Table 3).

**Figure 9.**
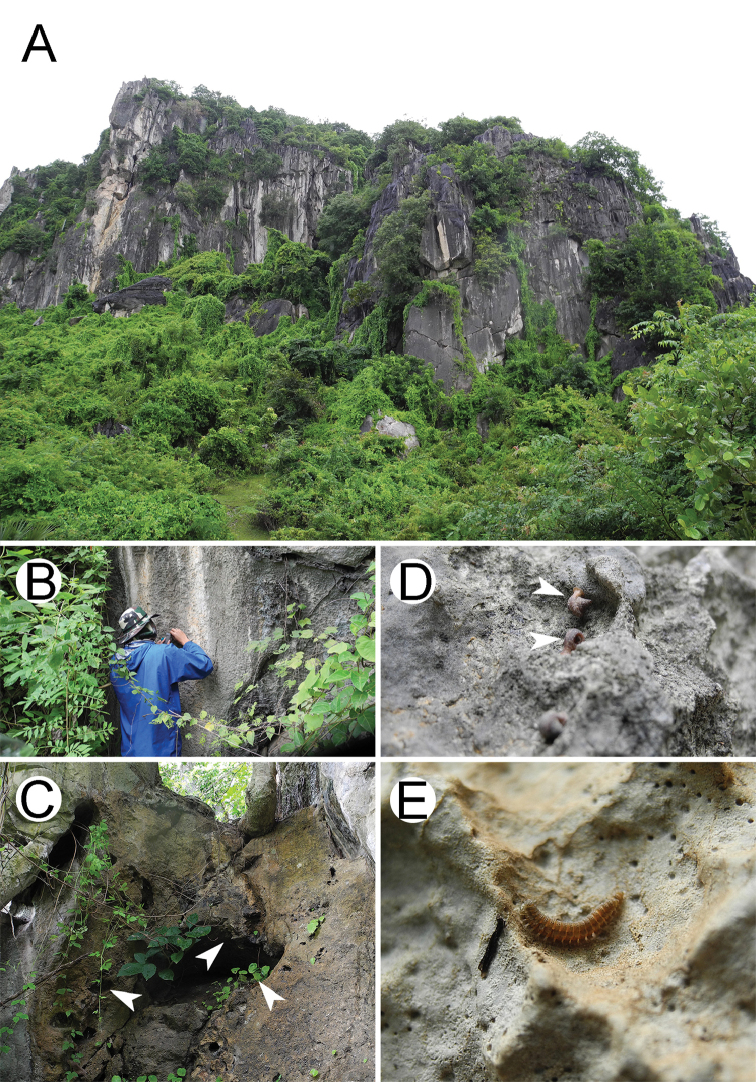
Habitat at the type locality of *Eutrichodesmus cambodiensis* sp. nov. **A** limestone outcrop **B, C** humid rock wall, sinkholes and crevices **D** co-occurrence of the new species with microsnail, *Hypselostoma cambodjense* Benthem Jutting, 1962 and **E** mating couple (male on top).

**Figure 10.**
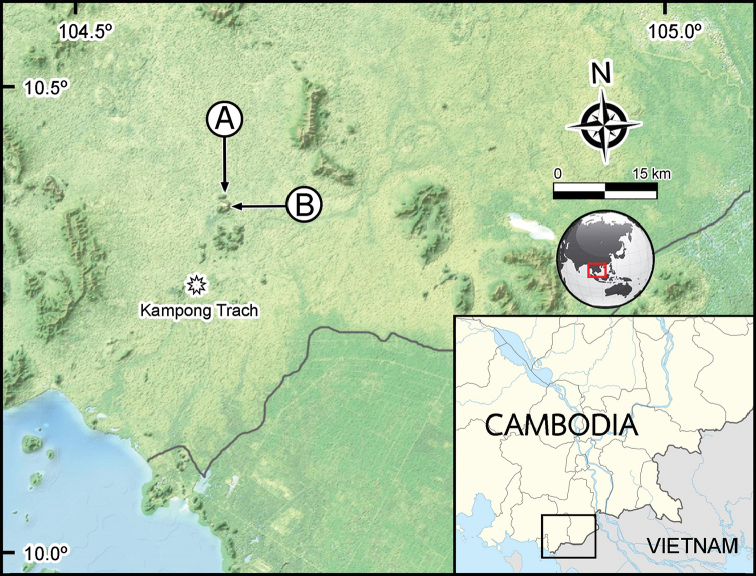
Known distribution of the *Eutrichodesmus cambodiensis* sp. nov. **A** limestone mount near Wat Phnom Small (type locality) **B** Prasat Phnom Totong.

### Key to species of *Eutrichodesmus* occurring in mainland Southeast Asia

From the previous records and including the new species described here, the genus *Eutrichodesmus* contains 55 known species from Japan (5), Taiwan (1), China (24), Vietnam (12), Laos (6), Cambodia (1), Thailand (2), Malaysia (2), Indonesia (1) and Vanautu (1), as detailed in Table 1. The new species shares most of the morphological characters to a bunch of species that are known to exist in Malaysia, Laos, Thailand and Vietnam. Here, we present an identification key, updated from the key of Golovacth et al. (2009b), for species of *Eutrichodesmus* occurring in mainland Southeast Asia (= 23 species).

**Table:** 

1	Ozopores visible, either absent or reduced (if present, appearing only on ring 17)	**2**
–	Ozopores normal, present at rings 5, 7, 9, 10, 12, 13 and 15–19	**4**
2	Body length ca. 6.0 mm. Metatergawith conspicuous and remarkable conical tubercles, each tubercle with several bacilliform setae and a long macroseta (Fig. 6C). Distofemoral process of gonopod telopodite quite short, lamelliform, triangular (Fig. 8B)	***E. gremialis***
–	Body length 7.5–9.0 mm. Metatergawith flattened or regular tubercles. Distofemoral process of gonopodal telopodite very long, digitiform or tube-like (Fig. 7G, K)	**3**
3	Gonopodal telopodite: distofemoral process bare; acropodite micropapillate at base, distally with two small teeth (Fig. 7G). Limbus crenulate	***E. deporatus***
–	Gonopodal telopodite: distofemoral process denticulate; acropodite not micropapillae at base, distally with one small tooth and a long digitiform lobe (Fig. 7K). Limbus microcrenulate	***E. paraster***
4	Metaterga with mid-dorsal projections or crests, conspicuous, present in mid-body or posteriormost body rings	**5**
–	Metaterga either with only slightly elevated and inconspicuous mid-dorsal tubercles in the last two rings or lacking conspicuous mid-dorsal projections or crests	**14**
5	Adult with 20 body rings	**6**
–	Adult with 19 body rings	**11**
6	Mid-dorsal projections on metaterga 16–19 or 17–19. Ozopores opening on evident porosteles in most body rings	***E. armatocaudatus***
–	Mid-dorsal projections on metaterga 3–19 or 4–19 or 5–19. Ozopores opening on a lobe of paraterga either without porosteles or porosteles appearing on posteriormost rings	**7**
7	Mid-dorsal projection on ring 5 inclined anteriorly at about 45°, directed anteriad or anterodorsad, not subvertical	***E. reclinatus***
–	Mid-dorsal projection on ring 5 nearly vertical (straight), directed dorsad or dorsoposteriad	**8**
8	Metaterga 3–19 with mid-dorsal projections. Acropodite quite short, less than one-third the length of telopodite (Fig. 7F).	***E. cavernicola***
–	Metaterga 4–19 or 5–19 with mid-dorsal projections. Acopodite quite long, almost half the length of telopodite (Fig. 7B, J, M).	**9**
9	Body length 12.0–14.0 mm. Acropodite without lobe; tip simple, unbranched (Fig. 7B)	***E. aster***
–	Body length 8.0–10.0 mm. Acropodite with a lobe or flange or micropapillate processes, forming a trifid or bifid tip (Fig. 7J, M)	**10**
10	Paraterga quite short (Fig. 5P). Distofemoral process longer, curved down. Tip of acropodite bifid (Fig. 7M)	***E. steineri***
–	Paraterga very long (Fig. 5J). Distofemoral process shorter, curved upwards. Tip of acropodite trifid (Fig. 7J)	***E. macclurei***
11	Distofemoral process absent (Fig. 7D)	***E. astriproximus***
–	Distofemoral precess present, short or long (Figs 7C, E, N)	**12**
12	Distofemoral process shorter, rudimentary and prong-shaped; acropodite with neither a hairpad nor a hairy pulvillus (Fig. 7C). Limbus clearly crenulate	***E. asteroides***
–	Distofemoral process quite long and slender, a digitiform; acropodite with a hairpad or a hairy pulvillus (Fig. 7E, N). Limbus microcrenulate	**13**
13	Acropodite quite short, less than half the length of prefemur; without lobe at about midway; base enlarged, fringed, velum-like (Fig. 7E)	***E. astrisimilis***
–	Acropodite long, about half the length of prefemur; lamelliform; with a mesal lobe at about midway; base normal, neither enlarged nor fringed (Fig. 7N)	***E. subasteroides***
14	Distofemoral process of telopodite short, inconspicuous, apparent and visible only in mesal view (Figs 7T; 8A, C)	**15**
–	Distofemoral process of telopodite very long, conspicuous, easily seen in many views (Figs 7I, S, V, W; 8J, L, P)	**17**
15	Body with complete volvation, larger, length 12.0–13.0 mm. Metatergawith three rows of tubercles, mixostictic; with slightly elevated mid-dorsal tubercles in last two rings. Tip of acropodite branched, forming a bifid tip and a lamella. Solenomere fused with acropodite (Fig. 8A)	***E. filisetiger***
–	Body with incomplete volvation, smaller, length 5.5–8.0 mm. Metatergawith three rows of tubercles, isostictic or nearly so; without a little elevated mid-dorsal tubercles in last two rings. Tip of acropodite unbranched, terminating in a dentiform tip. Solenomere not fused with acropodite, conspicuous, digitiform (Figs 7T; 8C)	**16**
16	Body grey to blackish. Paraterga shorter (Fig. 6D). Ozopores opening on evident porosteles. Limbus spinulate (comb-like), spinicles sharp and very much (more than twice) longer than broad. Solenomere not papillate, with a hairpad (Fig. 8C)	***E. griseus***
–	Body greyish-brown or light brown. Paraterga quite longer (Fig. 5W). Ozopores opening on a lobe on paraterga without porostele. Limbus crenulate, lobules slightly (less than twice) longer than broad, tip of each lobule round. Solenomere papillate, without hairpad (Figs 4; 7T)	***E. cambodiensis* sp. nov.**
17	Mid-dorsal tubercles on metaterga 18 and 19 slightly elevated and particularly evident	***E. multilobatus***
–	Mid-dorsal tubercles on metaterga 18 and 19 normal, neither elevated nor evident (e.g. Figs 2A, G; 3C)	**18**
18	Distofemoral process bare, surface smooth (Fig. 7W)	***E. demangei***
–	Distofemoral process denticulate (Figs 7S, V; 8J, L, P)	**19**
19	Body larger, length 9.0–10.0 mm. Rows of tubercles on metaterga isostictic in both longitudinal and transverse directions. Acropodite with a lobe at base	***E. regularis***
–	Body smaller, length 5.0–5.3 mm. Rows of tubercles on metaterga mixostictic. Acropodite without lobe at base	**20**
20	Gonopod : telopodite very simple; tip of acropodite with neither a conspicuous lobe nor a tooth (Fig. 7S). Paraterga very small (Fig. 5V)	***E. basalis***
–	Gonopod : telopodite not so simple; tip of acropodite with a conspicuous lobe or tooth (Figs 7V; 8J, L). Paraterga large (Figs 5Y; 6K, M)	**21**
21	Body volvation incomplete. Distal region of acropodite with a tooth and a lobe (Fig. 8L)	***E. parvus***
–	Body volvation complete. Distal region of acropodite with either a tooth or a lobe (Figs 7V; 8J)	**22**
22	Tip of acropodite unciform, slender. Seminal groove opening at about midway of acropodite; with neither a hairpad nor a hairy pulvillus (Fig. 8J). Limbus clearly microcrenulate.	***E. nadan***
–	Tip of acropodite neither unciform nor slender. Seminal groove opening near tip of acropodite; with a hairy pulvillus (Fig. 7V). Limbus irregularly crenulate.	***E. curticornis***

## Discussion

Prior to this study, the millipede fauna of Cambodia consisted of only 23 species, over half of which were described, based on a few specimens from just a handful of locations (Attems 1938, 1953; Likhitrakarn et al. 2015, 2020; Golovatch 2018). Amongst these, only the polydesmidan families Paradoxosomatidae and Cryptodesmidae have been known to occur in that country (Likhitrakarn et al. 2015; Golovatch 2018). No micropolydesmoid representative of the family Haplodesmidae has hitherto been reported from Cambodia. This situation is partly remedied herewith by the discovery and description of *E. cambodiensis* sp. nov.

*Eutrichodesmus cambodiensis* sp. nov. was exclusively found in isolated limestone habitats at or around caves. Based on its apparently highly restricted distribution, the new species can soundly be considered as endemic not only to Cambodia, but also to the Kampong Trach karst. As it is evident from Table 3, almost all *Eutrichodesmus* species have been found and collected from just one or a few locations confined to small areas. This strongly suggests that they are likely to be endemic to the respective areas and that further micropolydesmoids are most likely to be found in Cambodia.

The new species seems to have partial associations with caves, but it does not tend to show a troglomorphy syndrome because it is pigmented, has no hypertrophied appendages and no specimens have been found living inside the deep cave. Accordingly, this is no troglobite. Nevertheless, certain troglomorphic traits have been suggested in several species of *Eutrichodesmus*. For example, of the 55 currently known species, 24 are endemic to China alone and over half of these as troglobites, which is definitely a strong concentration of species in the region (Liu and Wynne 2019; Golovatch and Liu 2020). The same tendency to troglomorphy is also marked in most species known from other countries (Hoffman 1977a, 1977b, 1982a; Golovatch et al. 2009a, 2016; Liu et al. 2017).

The mostly thorough work by previous authors has provided sufficiently detailed information on important taxonomic characters that have allowed for species comparisons across *Eutrichodesmus* to be conducted (Golovatch et al. 2009a, 2009b, 2010, 2015, 2016; Liu et al. 2017). Two species groups of *Eutrichodesmus* are recognisable to account for the wide variety of morphological traits. The “*peculiaris*-group” was established by Golovatch et al. (2010) and currently accommodates seven species, while 46 species are harboured together in the second, “*demangei*-group” proposed in this study. Remarkably, the details of gonopodal conformation and the number and arrangement of the rows of tubercles on metaterga support the species assignments to either group. However, although these traits tend to be reliable, two species (*E. communicans* and *E. reductus*) could not satisfactorily be assigned into either group and thus remain ungrouped (see Table 3). The new species, *E. cambodiensis* sp. nov., shows all of its unique characters that are in agreement with its placement in the *demangei*-group.

In addition to gonopodal morphology, many families of the order Polydesmida prove the great utility of certain surface structures and some other peripheral characters for family- or genus-level classifications (Simonsen 1990; Shear 2008; Mesibov 2009; Akkari and Enghoff 2011). Within *Eutrichodesmus*, the basic knowledge of periperhal characters for a few old species is very scarce, with no available SEM images. Hence, this requires special attention in the future. With its 55 described species widely distributed in many countries, *Eutrichodesmus* seems to be the largest group of the micropolydesmoid family Haplodesmidae, but their phylogenetic relationships still remain unknown. Very little can be said about the presumed relationship between the Haplodesmidae and its recent synonym Doratodesmidae, as this synonymy is based solely on a few morphological characters (Hoffman 1982a, 1982b; Simonsen 1990; Golovatch 2009a). The further cladistic analysis and a molecular study are the obvious choices to improve the taxonomy by shedding further light on the group’s diversity in these millipedes.

The finding of a new *Eutrichodesmus* species in Cambodia fills in the gap in the distribution of the group across the eastern part of mainland Southeast Asia. As demonstrated recently by the discoveries of micropolydesmoids and other millipedes in the adjacent areas (Likhitrakarn et al. 2010, 2011, 2019; Golovatch et al. 2016, Liu et al. 2014, 2016, 2017; Golovatch 2018; Srisonchai et al. 2018) with respect to the unexplored and isolated limestone in Cambodia, Malaysia, Myanmar, Laos and Thailand, no doubt further new species remain to be discovered. It is hoped that this work will be a useful contribution to the ongoing process of documenting the diversity of Diplopoda in Cambodia and promote further studies on these remarkable creatures.

## Supplementary Material

XML Treatment for Eutrichodesmus

XML Treatment for Eutrichodesmus cambodiensis
